# Fast & fuelious: the malate–aspartate shuttle in brown adipocyte lipid metabolism

**DOI:** 10.1111/febs.70557

**Published:** 2026-04-24

**Authors:** Lukas Blaas, Alexander Bartelt

**Affiliations:** ^1^ Chair of Translational Nutritional Medicine, TUM School of Life Sciences Technical University of Munich Freising Germany; ^2^ Else Kröner Fresenius Center for Nutritional Medicine Germany; ^3^ Institute for Cardiovascular Prevention (IPEK), Faculty of Medicine, Ludwig‐Maximilians‐Universität München Munich Germany; ^4^ Institute for Diabetes and Cancer (IDC), Helmholtz Center Munich Neuherberg Germany; ^5^ German Center for Cardiovascular Research Partner Site Munich Heart Alliance, Ludwig‐Maximilians‐Universität München Munich Germany; ^6^ German Center for Diabetes Research Neuherberg Germany

**Keywords:** brown adipose tissue, lipid mobilization, malate–aspartate shuttle, metabolic redox balance, thermogenesis

## Abstract

Brown adipose tissue (BAT) produces heat in response to cold exposure, for which it relies on the coordination of aerobic and anaerobic metabolism. However, how reaction intermediates connect these two essential pathways is unclear. In this issue of *The FEBS Journal*, Veliova *et al.*, report that the malate–aspartate shuttle (MAS) supports norepinephrine‐induced lipolysis in brown adipocytes. Disruption of MAS during adrenergic activation impairs lipolysis without reducing respiration. These findings indicate that cytosolic redox balance influences thermogenic metabolism. By linking NAD+ regeneration to lipid metabolism, the study highlights the MAS as an important node coordinating metabolism, redox balance, and thermogenesis.

## Graphical abstract

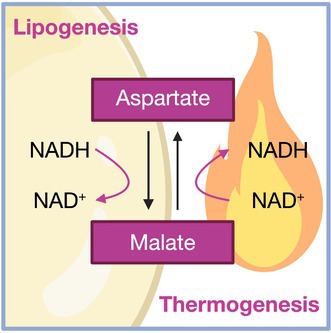

**Abbreviations**
AIFM2/FSP1: apoptosis‐inducing factor mitochondria‐associated 2
ARALAR1: aspartate–glutamate carrier 1
BAT: brown adipose tissue
GOT1: glutamic‐oxaloacetic transaminase 1
LD: lipid droplet
MAS: malate–aspartate shuttle
NE: norepinephrine
NEFA: non‐esterified fatty acids
OGC: oxoglutarate carrier
PKA: protein kinase A
UCP1: uncoupling protein 1

Non‐shivering thermogenesis is the process by which brown adipose tissue (BAT) dissipates chemical energy as heat, which is linked to high oxygen consumption and overall increased metabolism. The associated caloric demand is met through the continuous supply of BAT with fatty acids derived from intracellular and vascular lipolysis, as well as from glucose [1, 2, 3]. While non‐esterified fatty acids (NEFA) directly activate uncoupling protein 1 (UCP1) and serve as substrates for mitochondrial oxidation, BAT also sustains glycolytic flux. Even though BAT metabolism is predominantly lipid‐driven, especially under adrenergic conditions [4], restricting glycolysis directly impairs respiration [5]. Glycolysis generates cytosolic NADH, but this process requires the continuous regeneration of NAD^+^. How glycolysis, the Krebs cycle, and oxidative phosphorylation are coordinated during thermogenesis is still insufficiently understood.

Cytosolic redox equivalents need to be constantly regenerated during thermogenesis. This is physiologically relevant, as BAT depends on effective glycolytic and lipolytic flux geared towards mitochondrial oxidation. Failure to maintain redox balance often results in ferroptotic cell death [6]. The malate–aspartate shuttle (MAS) utilizes the metabolic intermediates malate, oxaloacetate, and aspartate as shuttling substrates, thus forming a regenerative cycle to balance the redox equivalents NAD^+^ and NADH [7]. This allows MAS to transfer electrons from cytosolic reactions to the inner membrane of the mitochondria, which supports oxidative phosphorylation. Previous studies have linked MAS to the redox network during thermogenesis [8, 9]: blocking mitochondrial pyruvate import engages MAS in brown adipocytes and promotes lipid cycling through re‐esterification and mobilization of NEFA [8]. As a result, cells increased their basal respiration, which is predominantly dependent on lipid oxidation. In addition, more recent work showed that glutamic oxaloacetic transaminase (GOT1) is a cold‐inducible enzyme that activates MAS, favoring NEFA over glucose usage in BAT [9].

Now, Veliova and colleagues extend the role of MAS in brown adipocyte function to the regulation of lipid metabolism [7]. Loss of the malate–alpha‐ketoglutarate (OGC) or the aspartate–glutamate carrier 1 (ARALAR1) resulted in an increased amount of small lipid droplets (LD) and blunted lipid mobilization after norepinephrine (NE) stimulation. Interestingly, despite this observation, overall oxygen consumption appeared largely preserved. The ability to maintain respiration when lipid mobilization is limited suggests that substrate accessibility and respiratory capacity can be regulated as distinct layers of BAT physiology. *In vivo*, BAT is a highly dynamic and complex metabolic tissue, as it simultaneously supports high catabolic metabolism as well as anabolic processes such as *de novo* lipogenesis [10]. Thermogenesis is also associated with the regulation of both coupled and uncoupled respiration [11]. However, although cells appear to employ alternative systems to maintain their redox balance and buffer the broad physiological function of the respiratory chain, thermogenesis can be constrained by limiting fuel access. In that sense, this new perspective moves the MAS further upstream in the thermogenic hierarchy, from only coordinating fuel preference to also dictating substrate availability.

During thermogenic activation, BAT likely relies on multiple redox‐buffering systems that operate in parallel and support distinct aspects of thermogenic metabolism. The authors of the study primarily discuss the glycerol‐3‐phosphate shuttle as the most plausible compensatory route that preserves redox balance and oxygen consumption when MAS is impaired [7]. While this might result in rerouting of metabolism towards re‐esterification/lipid cycling, it may not fully capture the underlying compensation of the preserved respiratory phenotype. Importantly, MAS‐deficient brown adipocytes seem to increase their mitochondrial area, likely through mitochondrial biogenesis. Hence, once respiration is normalized to mitochondrial content, MAS‐deficient cells have reduced oxygen consumption. To assess whether this is due to impaired respiratory chain efficiency or limited substrate availability, measurements of isolated mitochondria respiration, altered supercomplex composition, or membrane potential would be informative. A broader BAT‐related perspective might also include mechanisms such as elevated apoptosis‐inducing factor 2 (AIFM2) activity, a ferroptosis‐regulating NADH‐oxidizing enzyme, which has been shown to support glycolysis and oxygen consumption during thermogenic activation of BAT [12]. Interestingly, AIFM2, also known as FSP1, is spatially located at the lipid droplet and, upon cold stimulation, relocates inside of mitochondria. This places it directly at the intersection of lipid metabolism and oxidation, perhaps compensating for the lack of MAS by buffering NAD^+^/NADH homeostasis. Recent work has also highlighted mitochondrial NADP(H) as a parallel layer of redox balance that sustains oxidative competence through mitochondrial fatty acid synthesis‐dependent lipoylation of key mitochondrial enzymes [13]. This raises the possibility that preserved respiration in MAS‐deficient brown adipocytes may reflect compensation not only by alternative cytosolic shuttles but also by matrix‐dependent pathways.

The physiological implications of the observed phenotype may not only depend on the overall rate of thermogenesis but also on how efficiently NEFA are translocated to mitochondria during adrenergic activation. Organelles in brown adipocytes form an integrated metabolic network architecture in which mitochondria physically and functionally associate with lipid droplets. Impairment of MAS activity appears to predominantly influence adrenergic lipid droplet remodeling and blunt lipid mobilization. There are several potential explanations for the observed phenotype: First, a defect in NE‐induced lipase‐mediated lipolysis. Second, changes in NEFA re‐esterification via diacylglycerol O‐acyltransferases and lipid cycling might lead to altered LD structure and confer resistance to lipolytic stimuli. Thus, perturbation of MAS may alter LD morphology by disrupting the balance between lipolysis and LD synthesis [14]. This might result in impaired routing of NEFA from LDs to mitochondria, implying that they are instead retained in LDs or redirected between storage and reesterification, possibly accumulating smaller LDs. Lastly, particularly in BAT, a substantial fraction of mitochondria physically interacts with LD, forming LD‐associated or peridroplet mitochondrial populations. These specialized mitochondria represent a segregated population with unique structure and function. It has been postulated that these support triacylglyceride synthesis and LD expansion rather than fueling maximal beta oxidation [15]. During thermogenic activation, their oxidative capacity is comparatively low, as they depart from LDs. As the lipolytic defect in MAS deficiency emerges only under NE stimulation, this might indicate that MAS is also enriched in these specialized mitochondria, which remains to be determined. Taken together, the impairment resulting from limited MAS activity might not merely be reduced lipolysis, but rather a disturbance of the triacylglyceride pool being assembled and mobilized during the complex metabolic processes activated during BAT thermogenesis (Fig. 1).

**Fig. 1:**
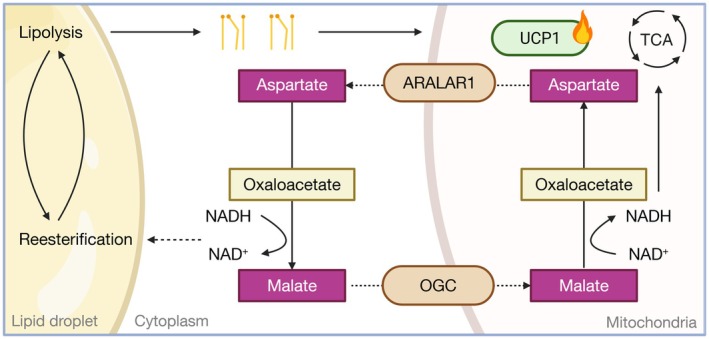
The malate–aspartate shuttle links cytosolic redox balance to lipid metabolism and thermogenic capacity in brown adipocytes. The malate–aspartate shuttle (MAS) uses the intermediate substrates oxaloacetate, malate, and aspartate to effectively transfer reducing equivalents from cytosolic NADH into mitochondria. Utilizing the mitochondrial transporters malate‐alpha‐ketoglutarate (OGC) and aspartate–glutamate carrier 1 (ARALAR1), MAS supports continued cytosolic NAD^+^ regeneration during glycolysis and likely influences the balance between lipolysis and re‐esterification in BAT. By connecting cytosolic redox homeostasis to mitochondrial metabolism, MAS appears to regulate thermogenic fuel availability upstream of substrate oxidation.

An important limitation of the current work is that its central findings are derived primarily from NE‐stimulated cultured primary brown adipocytes, which cannot fully capture the physiological complexity of cold or acute adrenergic activation *in vivo*. Whether MAS similarly constrains BAT function during chronic cold exposure or fed/fasted conditions remains unresolved, particularly as BAT fuel usage may shift depending on nutrient availability [2, 3]. *In vivo*, thermogenesis depends on intracellular lipolysis, sympathetic tone, substrate delivery from the circulation, and compensatory mechanisms that may buffer local defects in redox balance or fuel mobilization. Future studies will be required to determine whether MAS disruption via ARALAR1 and OGC alters cold tolerance, whole‐body energy expenditure, or substrate handling *in vivo*.

In conclusion, MAS is critical for efficient NE‐stimulated lipid mobilization in brown adipocytes. Disrupting MAS function alters LD morphology and blunts lipolysis while leaving respiratory output largely preserved. Future studies will be required to determine how the MAS interacts with other redox‐balancing pathways and whether these mechanisms influence thermogenic capacity *in vivo*, especially under cold or nutrient stress. More broadly, the present findings suggest that metabolic shuttle systems may influence thermogenesis not only by supporting oxidation but by shaping organelle communication and substrate preference.

## Conflicts of interest

The authors declare no conflicts of interest.

## Author contributions

LB prepared the manuscript and figure with input from AB.

## References

[1] Giroud M , Jodeleit H , Prentice KJ & Bartelt A (2022) Adipocyte function and the development of cardiometabolic disease. J Physiol 600, 1189–1208. 34555180 10.1113/JP281979

[2] Lemmer IL & Bartelt A (2023) Brown fat has a sweet tooth. Nat Metab 5, 1080–1081. 37337121 10.1038/s42255-023-00824-9

[3] Park G , Haley JA , Le J , Jung SM , Fitzgibbons TP , Korobkina ED , Li H , Fluharty SM , Chen Q , Spinelli JB *et al*. (2023) Quantitative analysis of metabolic fluxes in brown fat and skeletal muscle during thermogenesis. Nat Metab 5, 1204–1220. 37337122 10.1038/s42255-023-00825-8 PMC10696589

[4] Ouellet V , Labbé SM , Blondin DP , Phoenix S , Guérin B , Haman F , Turcotte EE , Richard D & Carpentier AC (2012) Brown adipose tissue oxidative metabolism contributes to energy expenditure during acute cold exposure in humans. J Clin Investig 122, 545–552. 22269323 10.1172/JCI60433 PMC3266793

[5] Winther S , Isidor MS , Basse AL , Skjoldborg N , Cheung A , Quistorff B & Hansen JB (2018) Restricting glycolysis impairs brown adipocyte glucose and oxygen consumption. Am J Physiol Endocrinol Metab 314, E214–E223. 29118013 10.1152/ajpendo.00218.2017

[6] Kotschi S , Jung A , Willemsen N , Ofoghi A , Proneth B , Conrad M & Bartelt A (2022) NFE2L1‐mediated proteasome function protects from ferroptosis. Mol Metab 57, 101436. 34999280 10.1016/j.molmet.2022.101436 PMC8814388

[7] Veliova M , Ferreira CM , Montales KP , Villalobos F , Brownstein AJ , Acín‐Pérez R , Ferreira GS , Jones AE , Stiles L , Divakaruni AS *et al*. (2026) The malate–aspartate shuttle supports thermogenic lipid mobilization in brown adipocytes. *FEBS J* 2025.08.04.667739. 10.1111/febs.70461 PMC13440609 41704162

[8] Veliova M , Ferreira CM , Benador IY , Jones AE , Mahdaviani K , Brownstein AJ , Desousa BR , Acín‐Pérez R , Petcherski A , Assali EA *et al*. (2020) Blocking mitochondrial pyruvate import in brown adipocytes induces energy wasting via lipid cycling. EMBO Rep 21, e49634. 33275313 10.15252/embr.201949634 PMC7726774

[9] Park CH , Park M , Kelly ME , Cheng H , Lee SR , Jang C & Chang JS (2025) Cold‐inducible GOT1 activates the malate‐aspartate shuttle in brown adipose tissue to support fuel preference for fatty acids. Cell Rep 44, 115888. 40540398 10.1016/j.celrep.2025.115888 PMC12294511

[10] Schlein C , Fischer AW , Sass F , Worthmann A , Tödter K , Jaeckstein MY , Behrens J , Lynes MD , Kiebish MA , Narain NR *et al*. (2021) Endogenous fatty acid synthesis drives Brown adipose tissue involution. Cell Rep 34, 108624. 33440156 10.1016/j.celrep.2020.108624 PMC8240962

[11] Brunetta HS , Jung AS , Valdivieso‐Rivera F , de Campos Zani SC , Guerra J , Furino VO , Francisco A , Berçot M , Moraes‐Vieira PM , Keipert S *et al*. (2024) IF1 is a cold‐regulated switch of ATP synthase hydrolytic activity to support thermogenesis in brown fat. EMBO J 43, 4870–4891. 39284909 10.1038/s44318-024-00215-0 PMC11535227

[12] Nguyen HP , Yi D , Lin F , Viscarra JA , Tabuchi C , Ngo K , Shin G , fai LAY , Wang Y & Sul HS (2020) Aifm2, a NADH oxidase, supports robust glycolysis and is required for cold‐ and diet‐induced thermogenesis. Mol Cell 77, 600–617. 31952989 10.1016/j.molcel.2019.12.002 PMC7031813

[13] Haumann F , Evangelakos I , Worthmann A , Liebold I , Kotschi S , Bischoff AT , Neuhofer CM , Schweizer M , Heine M , Büchner B *et al*. (2023) Mitochondrial fatty acid synthesis is essential for coordinated energy transformation. *medRxiv* 2023.04.03.23288010.

[14] Irshad Z , Dimitri F , Christian M & Zammit VA (2017) Diacylglycerol acyltransferase 2 links glucose utilization to fatty acid oxidation in the brown adipocytes. J Lipid Res 58, 15–30. 27836993 10.1194/jlr.M068197 PMC5234708

[15] Benador IY , Veliova M , Mahdaviani K , Petcherski A , Wikstrom JD , Assali EA , Acín‐Pérez R , Shum M , Oliveira MF , Cinti S *et al*. (2018) Mitochondria bound to lipid droplets have unique bioenergetics, composition, and dynamics that support lipid droplet expansion. Cell Metab 27, 869–885. 29617645 10.1016/j.cmet.2018.03.003 PMC5969538

